# Exome reanalysis and proteomic profiling identified *TRIP4* as a novel cause of cerebellar hypoplasia and spinal muscular atrophy (PCH1)

**DOI:** 10.1038/s41431-021-00851-8

**Published:** 2021-06-01

**Authors:** Ana Töpf, Angela Pyle, Helen Griffin, Leslie Matalonga, Katherine Schon, Solve-RD SNV-indel working group, Enzo Cohen, Isabel Cuesta, Daniel Danis, Anne-Sophie Denommé-Pichon, Yannis Duffourd, Christian Gilissen, Mridul Johari, Steven Laurie, Shuang Li, Isabelle Nelson, Ida Paramonov, Sophia Peters, Sivakumar Prasanth, Peter Robinson, Karolis Sablauskas, Marco Savarese, Wouter Steyaert, Joeri K. van der Velde, Antonio Vitobello, Solve-RD DITF-euroNMD, Jonathan Baets, Danique Beijer, Gisèle Bonne, Judith Cossins, Teresinha Evangelista, Alessandra Ferlini, Peter Hackman, Michael G. Hanna, Henry Houlden, Jarred Lau, Hanns Lochmüller, William L. Macken, Francesco Musacchia, Andres Nascimento, Daniel Natera-de Benito, Vincenzo Nigro, Giulio Piluso, Veronica Pini, Robert D. S. Pitceathly, Kiran Polavarapu, Pedro M. Rodriguez Cruz, Anna Sarkozy, Rita Selvatici, Rachel Thompson, Annalaura Torella, Bjarne Udd, Liedewei Van de Vondel, Jana Vandrovcova, Irina Zaharieva, Albert Sickmann, Ulrike Schara–Schmidt, Andreas Hentschel, Patrick F. Chinnery, Heike Kölbel, Andreas Roos, Rita Horvath

**Affiliations:** 1 John Walton Muscular Dystrophy Research Centre, Translational and Clinical Research Institute, Newcastle University and Newcastle Hospitals NHS Foundation Trust, Newcastle upon Tyne, UK; 2 Wellcome Centre for Mitochondrial Research, Translational and Clinical Research Institute, Newcastle University, Newcastle upon Tyne, UK; 3 Primary Immunodeficiency Group, Newcastle University Translational and Clinical Research Institute, Newcastle upon Tyne, UK; 4 CNAG‐CRG, Centre for Genomic Regulation (CRG), The Barcelona Institute of Science and Technology, Barcelona, Spain; 5 Department of Clinical Neurosciences, University of Cambridge, Cambridge, UK; 6 MRC Mitochondrial Biology Unit, Cambridge Biomedical Campus, Cambridge, UK; 7 Department of Bioanalytics, Leibniz-Institut für Analytische Wissenschaften-ISAS-e.V., Dortmund, Germany; 8 Department of Chemistry, College of Physical Sciences, University of Aberdeen, Aberdeen, Scotland UK; 9 Medizinische Proteom-Center (MPC), Medizinische Fakultät, Ruhr-Universität Bochum, Bochum, Germany; 10 Department of Pediatric Neurology, Developmental Neurology and Social Pediatrics, Children’s Hospital University of Essen, Essen, Germany; 11 Leibniz-Institut für Analytische Wissenschaften - ISAS - e.V., Dortmund, Germany; 12 Sorbonne Université, Inserm, Institut de Myologie, Centre de Recherche en Myologie, F-75013, Paris, France; 13 Instituto de Salud Carlos III, Madrid, Spain; 14 Jackson Laboratory for Genomic Medicine, Farmington, CT USA; 15 Inserm - University of Burgundy-Franche Comté, Dijon, France; 16 Dijon University Hospital, FHU-TRANSLAD, Dijon, France; 17 Department of Human Genetics, Radboud University Medical Center, Nijmegen, The Netherlands; 18 Radboud Institute for Molecular Life Sciences, Nijmegen, the Netherlands; 19 Folkhälsan Research Center, University of Helsinki, Helsinki, Finland; 20 Department of Genetics, Genomics Coordination Center, University Medical Center Groningen, University of Groningen, Groningen, The Netherlands; 21 Institute of Human Genetics, University of Bonn, Bonn, Germany; 22 Department of Neuromuscular Diseases, UCL Queen Square Institute of Neurology and The National Hospital for Neurology and Neurosurgery, London, UK; 23 Peripheral Neuropathy Research Group, University of Antwerp, Antwerp, Belgium; 24 Neuromuscular Reference Centre, Department of Neurology, Antwerp University Hospital, Antwerpen, Belgium; 25 Laboratory of Neuromuscular Pathology, Institute Born-Bunge, University of Antwerp, Antwerpen, Belgium; 26 Neuromuscular Disorders Group, NDCN, Weatherall Institute of Molecular Medicine, John Radcliffe Hospital, Oxford, UK; 27 Unit of Medical Genetics, Department of Medical Sciences, University of Ferrara, Ferrara, Italy; 28 Children’s Hospital of Eastern Ontario Research Institute, Ottawa, ON Canada; 29 Division of Neurology, Department of Medicine, The Ottawa Hospital, Ottawa, ON Canada; 30 Brain and Mind Research Institute, University of Ottawa, Ottawa, ON Canada; 31 Department of Neuropediatrics and Muscle Disorders, Faculty of Medicine, Medical Center – University of Freiburg, Freiburg, Germany; 32 Dipartimento di Medicina di Precisione, Università degli Studi della Campania “Luigi Vanvitelli”, Napoli, Italy; 33 Telethon Institute of Genetics and Medicine, Pozzuoli, Italy; 34 Neuromuscular Unit, Neuropaediatrics Department, Institut de Recerca Pediàtrica Hospital Sant Joan de Déu, CIBERER, Barcelona, Spain; 35 Dubowitz Neuromuscular Centre, UCL Great Ormond Street Hospital, London, UK; 36 Nuffield Department of Clinical Neurosciences, University of Oxford, Oxford, UK

**Keywords:** Genetics research, Next-generation sequencing

## Abstract

TRIP4 is one of the subunits of the transcriptional coregulator ASC-1, a ribonucleoprotein complex that participates in transcriptional coactivation and RNA processing events. Recessive variants in the *TRIP4* gene have been associated with spinal muscular atrophy with bone fractures as well as a severe form of congenital muscular dystrophy. Here we present the diagnostic journey of a patient with cerebellar hypoplasia and spinal muscular atrophy (PCH1) and congenital bone fractures. Initial exome sequencing analysis revealed no candidate variants. Reanalysis of the exome data by inclusion in the Solve-RD project resulted in the identification of a homozygous stop-gain variant in the *TRIP4* gene, previously reported as disease-causing. This highlights the importance of analysis reiteration and improved and updated bioinformatic pipelines. Proteomic profile of the patient’s fibroblasts showed altered RNA-processing and impaired exosome activity supporting the pathogenicity of the detected variant. In addition, we identified a novel genetic form of PCH1, further strengthening the link of this characteristic phenotype with altered RNA metabolism.

## Introduction

The thyroid receptor interacting protein 4, encoded by the *TRIP4* gene, is one of the four subunits of the transcriptional coregulator ASC-1. TRIP4, together with ASCC1, ASCC2 and ASCC3, form a ribonucleoprotein complex that participates in transcriptional coactivation and RNA processing events [1]. TRIP4 binds transcription factors, such as AP-1 and NF-kappa-B through its conserved cysteine rich Zn-chelating domain [2], whilst the C-terminal RNA-binding PUA domain is involved in RNA processing [3].

Recessive variants in *TRIP4* have been associated with two distinct phenotypes: spinal muscular atrophy with bone fractures (OMIM #616866; ref. [1]) and a severe form of congenital muscular dystrophy (CMD)(OMIM #617066; ref. [4, 5]). Interestingly, changes in another subunit of the transcriptional coregulator ASC-1, ASCC1, also result in a prenatal muscle weakness with arthrogryposis and congenital bone fractures [1, 6]. Since the first reports in 2016 describing the two associated phenotypes, only five additional CMD families have been reported [5]. Here we present the diagnostic journey of a patient with cerebellar hypoplasia, spinal muscular atrophy (PCH1-like) and congenital bone fractures, where we identified a homozygous stop-gain variant in the *TRIP4* gene; the ninth case reported world-wide.

## Subjects and methods

This study was approved by the Ethics Committee of University of Duisburg-Essen (19-9011-BO). All the involved subjects gave their written informed consent.

### Exome sequencing

DNA from the index case and his parents was subjected to whole exome sequencing (WES) using Nextera Rapid Exome Capture (Illumina)(Supplementary material). Exonic and splice site variants with a minor allele frequency of <1% in external databases (i.e. ExAC, 1000 genomes) were prioritized. In silico prediction tools such as Polyphen2 (http://genetics.bwh.harvard.edu/pph2/), CADD (https://cadd.gs.washington.edu/) and SIFT (http://sift.jcvi.org/) were used to assess pathogenicity.

### WES reanalysis

The fastq files for the index case and his parents were submitted to Solve-RD [7] through the Genome-Phenome Analysis Platform (GPAP, https://platform.rd-connect.eu/) as part of the European Reference Network for Neuromuscular Disease (ERN-EURO-NMD, https://ern-euro-nmd.eu/), and processed using the RD-Connect bioinformatics pipeline [8]. The reanalysis strategy carried out by the SNV-Indels Working Group [7, 9] implemented automated data filtration to specifically identify previously reported disease-causing variants (as annotated in ClinVar v.13-01-2020) from a candidate gene list of 594 genes known to be associated with NMD [9]. The identified variant has been submitted to ClinVar (SUB8878662; https://www.ncbi.nlm.nih.gov/clinvar).

### Mass spectrometry based proteomic analysis

Control and patient-derived fibroblasts were grown to a 70% confluency and mechanically harvested. Label free shotgun proteomic analysis was performed as previously described [10]. To interrogate protein-dysregulation pathways a Gene Ontology (GO) term analysis was performed. Proteomaps (www.proteomaps.net) was used to visualise affected cellular functions. The mass spectrometry proteomics data was submitted to the ProteomeXchange Consortium via the PRIDE (PXD023584; https://www.ebi.ac.uk/pride).

## Results

### Clinical findings

**Fig. 1:**
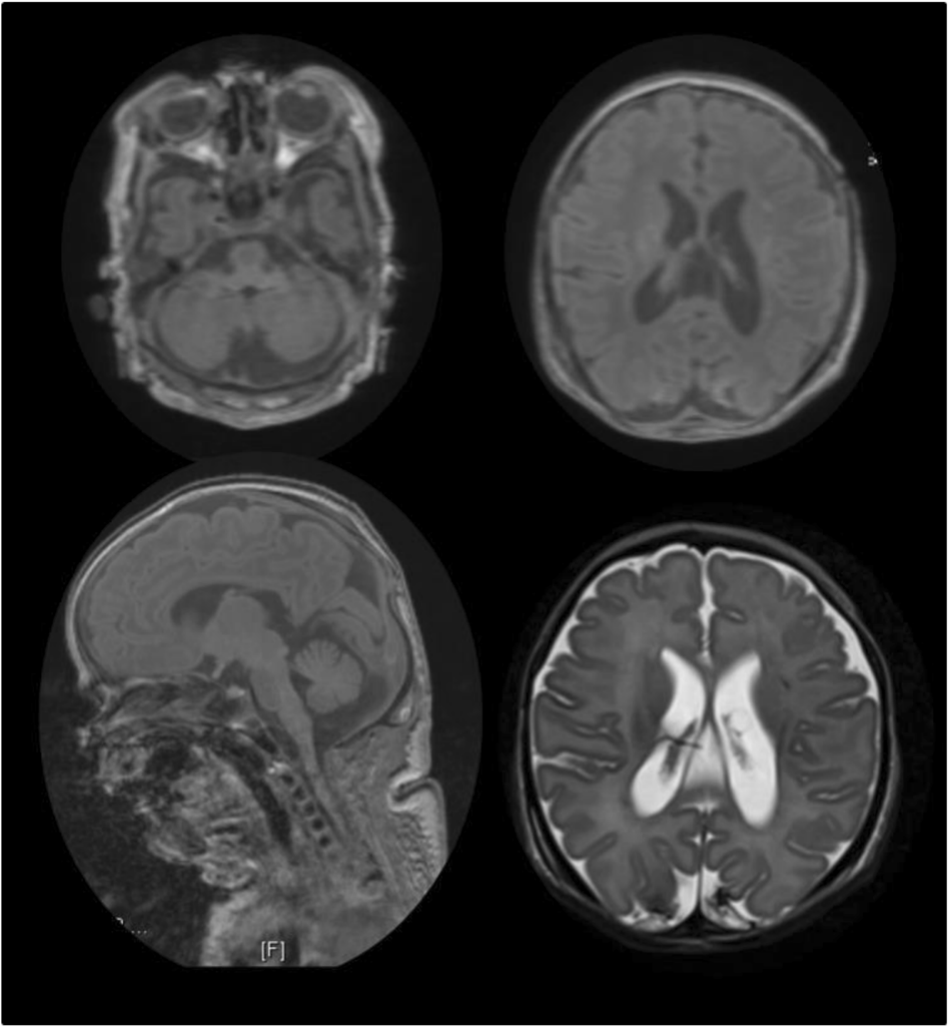
Magnetic resonance imaging (MRI). Cranial and thoracic MRI performed at the age of 6 weeks revealed a hypoplastic cerebellum, necrosis and haemorrhage of the occipital cortex, pronounced atrophy of skeletal musculature and confirmed bilateral pleural effusion.

The patient was the first child of consanguineous parents, born at 38 weeks of gestational age by Caesarean section (Apgar score: 5/5/5). Pregnancy was complicated by oligohydramnios, placental insufficiency and growth retardation. The newborn presented small for gestational age at birth (weight 2610 g [6th percentile], length 42 cm [<3th percentile], head circumference 34.5 cm [40th percentile]). Multiple contractures and intrauterine-originated fractures of both femurs and the right humerus were present at birth. He also showed respiratory insufficiency complicated by absence of spontaneous movements so that invasive ventilation was initiated. Clinical course was further characterised by pleural effusion and chylothorax, biliary calculi and soft tissue emphysema. Cranial and thoracic MRI performed at the age of 6 weeks revealed a hypoplastic cerebellum but normal pons, necrosis and haemorrhage of the occipital cortex, pronounced atrophy of skeletal musculature and confirmed bilateral pleural effusion (Fig. 1). After excluding 5q-SMA by genetic analysis of the *SMN1* gene, a vastus lateralis muscle biopsy was performed for further diagnostic evaluation, yet due to the massive atrophy, the number of intact fibres was too small to allow a valid pathological examination. Given the unfavourable prognosis, after consultation with the parents and the clinical ethical committee, invasive ventilation was terminated at the age of 8 weeks when the patient died. The combination of SMA and cerebellar hypoplasia resembled patients with disease-causing variants in components of the human RNA exosome [11].

### Initial genetic analysis

SMN1 and additional single-gene screening of *ACTA1*, *BIN1*, *DNM2*, *MTM1*, *RYR1*, *SEPN1*, *TNNT1*, *TPM2* and *TPM3* was negative. Trio exome sequencing was undertaken, with the first round of analysis taking place in 2017. Stringent filtering criteria retrieved no variants in known disease-causing or strong candidate genes.

### Reanalysis of exome data

The ‘low hanging fruit’ strategy implemented in 2020 by the SNV-Indels Working Group of Solve-RD [7, 9] meant only variants already known to be associated with disease were selected. Thus, a homozygous stop-gain variant [hg19:chr15:64698591 C > T; NM_016213.5:c.760 C > T; p.(Arg254*)] in the *TRIP4* gene was identified. This variant had been previously reported in three patients (from two families) with Prenatal Spinal Muscular Atrophy and Congenital Bone Fractures [1], proving to be a phenotypic match and final diagnosis to our patient.

### Proteomic findings

**Fig. 2:**
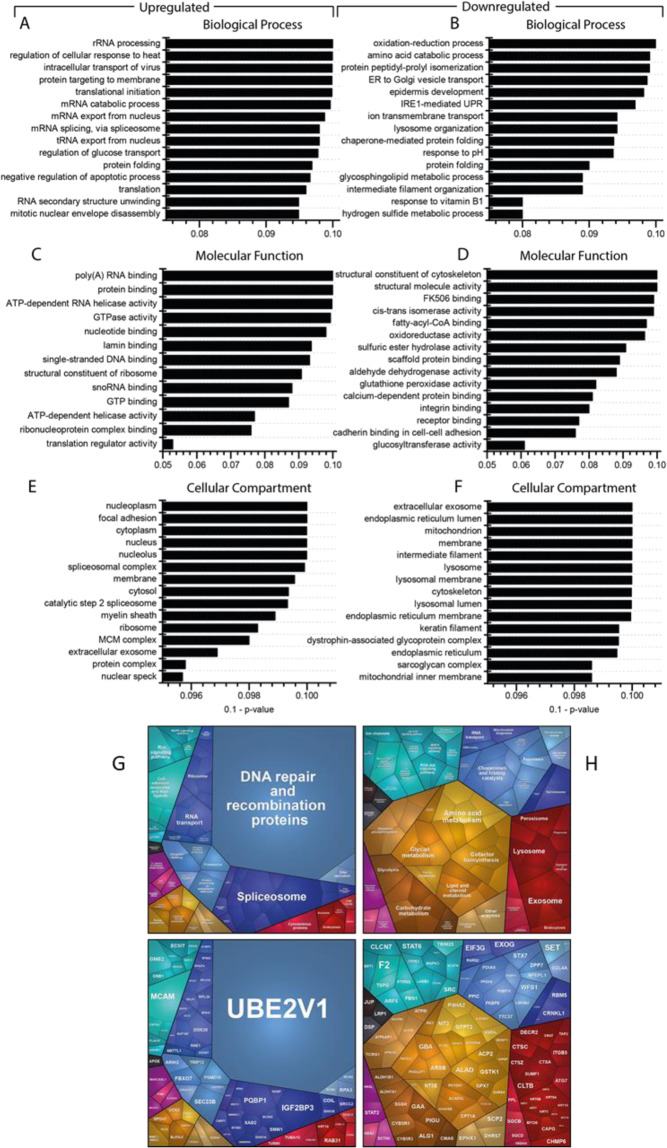
In silico studies of proteomic data. Gene Ontology (GO) term analysis was performed focussing on the up- and downregulated proteins separately: (**A**) “Biological Processes”revealed that the majority of increased proteins is involved in RNA processing, whereas most of decreased proteins (**B**) control the response to oxidative stress or are involved in protein folding. “Molecular Function” revealed that proteins involved in RNA-binding and modulating nucleotide binding are increased (**C**), and those involved in the cytoskeleton structure and the modulation of oxidative stress predominate amongst the downregulated (**D**). “Cellular Component” showed that the proteins upregulated in the patient-derived fibroblasts mostly belong to the nucleus (**E**) and the ER, the lysosome, mitochondria, and the cytoskeleton are downregulated (**F**). The proteomaps-based pathway analysis also focussed on increased and decreased proteins separately. **G** For increased proteins, it confirmed changes in RNA-transport and RNA-processing, and indicated an activation of the protein clearance machinery (Ubiquitin-mediated proteolysis via the proteasome) as well as altered signalling processes (Ras, ErbB and MAPK pathways). **H** For decreased proteins, indicated a vulnerability of cellular metabolism accompanied by perturbed signalling cascades (Jak-STAT, ErbB, MAPK, PI3K-AKT & NF-kappa-B pathways) and reduced lysosomal as well as exosome function.

To further validate the pathogenicity of the detected variant and to obtain biochemical insights into the underlying pathophysiology, a proteomic profile on patient-derived fibroblasts was carried out. Applying a data independent acquisition (DIA) approach, a total of 3558 proteins were quantified. Among these, 323 were significantly dysregulated (p-ANOVA ≤ 0.05): 143 (4.2%) and 180 (5.6%) proteins showed increased and decreased abundance, respectively. An intersection of dysregulated proteins with genetic variants revealed no significant correlations. Proteomaps and a GO term analysis revealed a dysregulation of diverse signaling cascades including the NF-kappa-B pathway. We also detected an increased abundance in proteins of the RNA-processing machinery, while decreased proteins suggested impaired exosomal activity (Fig. 2).

## Discussion

We describe the diagnostic journey of a patient presenting with a cerebellar hypoplasia and SMA (PCH1-like) phenotype with bone fractures, in whom the exome reanalysis using the RD-Connect/Solve-RD pipeline [8, 9] identified a homozygous stop-gain variant in the *TRIP4* gene. Intriguingly, the disease-causing variant was not found during the first round of analysis (circa 2017), even though coverage at this position was >40x. *TRIP4* was associated with disease only in 2016 [1, 4], however the initial WES analysis searched for candidate variants in the whole exome, and not just a gene list of known associated genes. Since the *TRIP4* variant is a highly deleterious homozygous variant, it seems unlikely that it would have not been picked as a strong candidate. In fact, by interrogating filtered VCF files we speculate that the variant must have been filtered out during the bioinformatics steps. This emphasises that reevaluation of legacy exome data should be performed not only by periodic reiteration of the analysis [12], but also by improved and updated bioinformatic pipelines, such as those implemented in Solve-RD [7–9].

*TRIP4* encodes a transcription coactivator which facilitates nuclear receptors-mediated transcription and ribosome-associated quality control in mammalian cells [13]. It has been also shown to play a role in transactivation of NF-kappa-B [2].

The combination of genomics, transcriptomics and proteomics is increasingly being used to correlate protein abundances with genetic variants, as well as to delineate pathways affected by the loss of a functional protein [14, 15]. Such approaches build on the assumption that protein interaction networks can be viewed as maps in which diseases can be identified with characteristic proteomic signatures [14, 16]. Tissues such as blood, skin or muscle biopsies provide a source of DNA, RNA and protein [14] suitable to study the molecular etiology of these diseases [10]. Here, we showed how proteomic profiling was carried out in a patient with PCH1-like disease with bone fractures to evaluate the pathogenicity of the detected variant and to obtain insights into the biochemical etiology. Our pathway-analyses of proteomic findings highlighted perturbations in RNA-processing and signaling processes including the NF-kappa-B pathway. We detected decrease in abundance of the RNA exosome complexes, in line with the clinical presentation of PCH1 [17], however no variant was detected in any of the known genes encoding subunits or accessory proteins of the human exosome complex. Although the TRIP4 protein was not detectable on the proteomics profile, alteration of other proteins resulted in a “proteomic signature”, highlighting the molecular consequences of the loss of TRIP4. The similar clinical presentation of patients with variants in TRIP4 and exosomal proteins further highlights that PCH1 is genetically heterogeneous, and linked with ribosome dysfunction and abnormal RNA processing [18].

### Clinical and biochemical synopsis

A neurological syndrome combining cerebellar hypoplasia and spinal motor muscular atrophy is already linked to exosomal protein variants expanding the list of human exosomopathies [11]. The RNA exosome represents a conserved multi-protein complex essential for gene expression via processing and degradation of mRNA [19]. Proteomic pathway analysis in a patient with a novel *TRIP4* variant revealed a dysregulation of RNA-processing and down-regulation of exosomal proteins. These findings suggest a common pathomechanism leading to the clinical manifestation of this neurological syndrome. However, comparative biochemical studies in in vitro or in vivo models are crucial to elucidate the precise molecular nature of the common pathophysiology.

## Supplementary information

Supplementary Material

Supplementary Table

Consortium Author List
