# Supplementary material for: Exome reanalysis and proteomic profiling identified TRIP4 as a novel cause of cerebellar hypoplasia and spinal muscular atrophy (PCH1)
Source: Eur J Hum Genet. 2021 Jun 1;29(9):1348–53. doi: 10.1038/s41431-021-00851-8 (PMC8440675; doi:10.1038/s41431-021-00851-8)
Supplement: Supplementary file 1 — Supplementary Material [file 41431_2021_851_MOESM1_ESM.docx]

# Supplementary Material

# Whole exome sequencing

Whole exome sequencing (WES) using Nextera Rapid Exome Capture (Illumina)(Supplementary material) and sequenced with 100 bp paired-end reads on an Illumina NextSeq500 platform. An in-house bioinformatics pipeline was applied to the raw FASTQ files. Briefly, PCR duplicates were removed using Fastuniq (Xu et al., 2012); Burrows-Wheeler Aligner (Li and Durbin, 2009) was used to align the resultant reads to the human reference genome (UCSC hg38); genetic variants were detected using Freebayes (Garrison and Marth, 2012) and functionally annotated using Annovar (Wang et al., 2010). Variants were prioritised if exonic or in a splice-site region with a minor allele frequency of <1% in external databases (i.e. ExAC, 1000 genomes). *In silico* prediction tools such as Polyphen 2 (http://genetics.bwh.harvard.edu/pph2/), CADD (Kirscher et al., 2014) and SIFT (http://sift.jcvi.org/) were used to assess pathogenicity.

References:

Xu H, Luo X, Qian J, Pang X, Song J, Qian G, Chen J, Chen S. (2012) FastUniq: a fast de novo duplicates removal tool for paired short reads. PLoS One, 7(12):e52249

Li H. and Durbin R. (2009) Fast and accurate short read alignment with Burrows-Wheeler Transform. Bioinformatics, 25:1754-60. Garrison E, Marth G. Haplotype-based variant detection from short-read sequencing. arXiv preprint arXiv:1207.3907 [q-bio.GN] 2012

Wang K, Li M, Hakonarson H. ANNOVAR: Functional annotation of genetic variants from next-generation sequencing data Nucleic Acids Research, 38:e164, 2010

Kirscher M, Witten DM, Jain P, O’Roak BJ, Cooper GM, Shendure J. (2014) A general framework for estimating the relative pathogenicity of human genetic variants. Nat Genet (3):310-5.
