# Supplementary material for: Exome reanalysis and proteomic profiling identified TRIP4 as a novel cause of cerebellar hypoplasia and spinal muscular atrophy (PCH1)
Source: Eur J Hum Genet. 2021 Jun 1;29(9):1348–53. doi: 10.1038/s41431-021-00851-8 (PMC8440675; doi:10.1038/s41431-021-00851-8)
Supplement: Supplementary file 3 — Consortium Author List [file 41431_2021_851_MOESM3_ESM.docx]

**Solve-RD SNV-indel working group**

Leslie Matalonga^4^*, Enzo Cohen^12^, Isabel Cuesta^13^, Daniel Danis^14^, Anne-Sophie Denommé-Pichon^15^, Yannis Duffourd^15,16^, Christian Gilissen^17,18^, Mridul Johari^19^, Steven Laurie^4,^ Shuang Li^20^, Isabelle Nelson^12^, Ida Paramonov^4^, Sophia Peters^21^, Sivakumar Prasanth^22^, Peter Robinson^14^, Karolis Sablauskas^17,18^, Marco Savarese^19^, Wouter Steyaert^17,18^, Ana Töpf^1^, Joeri K van der Velde^20^ and Antonio Vitobello^15^.

**Solve-RD-DITF-euroNMD**

Ana Töpf^1^*, Jonathan Baets^24,25,26^, Danique Beijer^24,25^, Gisèle Bonne^12^, Enzo Cohen^12^, Judith Cossins^27^, Teresinha Evangelista^12^, Alessandra Ferlini^28^, Peter Hackman^19^ , Michael G Hanna^22^, Rita Horvath^5^, Henry Houlden^22^, Mridul Johari^19,^ Jarred Lau^29,^ Hanns Lochmüller^4,29,30,31,32^, William L Macken ^22^, Francesco Musacchia^33,34^, Andres Nascimento^35,^ Daniel Natera-de Benito ^35^, Vincenzo Nigro ^33,34,^ Giulio Piluso^33^, Veronica Pini^36^, Robert DS Pitceathly^22^, Kiran Polavarapu^29,32^, Pedro M Rodriguez Cruz^27,37^, Anna Sarkozy^36^, Marco Savarese^19^, Rita Selvatici^28^, Rachel Thompson^29^, Annalaura Torella ^33,34^, Bjarne Udd^19^, Liedewei Van de Vondel^24,25^, Jana Vandrovcova^22^ and Irina Zaharieva^36^.

**Affiliations**

12 Sorbonne Université, INSERM, Center of Research in Myology, Paris, France.

13 Instituto de Salud Carlos III, Madrid, Spain.

14 Jackson Laboratory for Genomic Medicine, Farmington, USA.

15 Inserm - University of Burgundy-Franche Comté, Dijon, France.

16 Dijon University Hospital, FHU-TRANSLAD, Dijon, France.

17 Department of Human Genetics, Radboud University Medical Center, Nijmegen, The Netherlands.

18 Radboud Institute for Molecular Life Sciences, Nijmegen, the Netherlands.

19 Folkhälsan Research Center, University of Helsinki and Tampere Neuromuscular Center, Finland.

20 Department of Genetics, Genomics Coordination Center, University Medical Center Groningen, University of Groningen, Groningen, The Netherlands.

21 Institute of Human Genetics, University of Bonn, Bonn, Germany.

22 Department of Neuromuscular Diseases, UCL Queen Square Institute of Neurology and The National Hospital for Neurology and Neurosurgery, London, UK.

23 John Walton Muscular Dystrophy Research Centre, Translational and Clinical Research Institute, Newcastle University and Newcastle Hospitals NHS Foundation Trust, Newcastle upon Tyne, UK.

24 Translational Neurosciences, Faculty of Medicine and Health Sciences, UAntwerpen, Antwerp, Belgium

25 Laboratory of Neuromuscular Pathology, Institute Born-Bunge, University of Antwerp, Antwerpen, Belgium

26 Neuromuscular Reference Centre, Department of Neurology, Antwerp University Hospital, Antwerpen, Belgium

27 Neuromuscular Disorders Group, NDCN, Weatherall Institute of Molecular Medicine, John Radcliffe Hospital, Oxford, UK.

28 Unit of Medical Genetics, Department of Medical Sciences, University of Ferrara, Italy.

29 Children's Hospital of Eastern Ontario Research Institute, Ottawa, Canada.

30 Division of Neurology, Department of Medicine, The Ottawa Hospital, Ottawa, Canada.

31 Brain and Mind Research Institute, University of Ottawa, Ottawa, Canada.

32 Department of Neuropediatrics and Muscle Disorders, Medical Center – University of Freiburg, Faculty of Medicine, Freiburg, Germany.

33 Dipartimento di Medicina di Precisione, Università degli Studi della Campania "Luigi Vanvitelli," Napoli, Italy.

34 Telethon Institute of Genetics and Medicine, Pozzuoli, Italy.

35 Neuromuscular Unit, Neuropaediatrics Department, Institut de Recerca Pediàtrica Hospital Sant Joan de Déu, CIBERER, Barcelona, Spain.

36 Dubowitz Neuromuscular Centre, UCL Great Ormond Street Hospital, London, UK.

37 Nuffield Department of Clinical Neurosciences, University of Oxford, UK.
